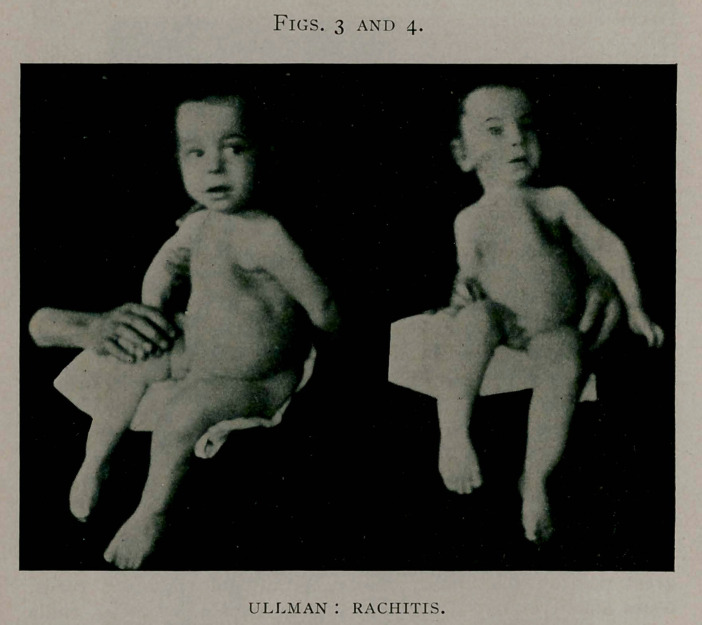# Report of Three Cases (1) Pseudoleukemia, (2) Elephantiasis, (3) Rachitis with Marked Thoracic Deformity in a Child Fourteen Months Old

**Published:** 1905-08

**Authors:** Julius Ullman

**Affiliations:** Instructor in clinical medicine, Medical Department University of Buffalo; attending physician German Hospital and University of Buffalo Dispensary. 400 Franklin Street


### CLINICAL REPORTS.

### Report of Three Cases (1) Pseudoleukemia, (2) Elephantiasis, (3) Rachitis with Marked Thoracic Deformity in a Child Fourteen Months Old.

By JULIUS ULLMAN, M. D.,
Instructor in clinical medicine, Medical Department University of Buffalo; attending physician German Hospital and University of Buffalo Dispensary.

IN THE July, 1901, number of the Buffalo Medical Journal, the writer illustrated two cases of splenomegaly in the course of malaria; the one, a recent estivoautumnal malaria; the other, a splenic anemia, probably of malarial origin. The case, which is reported in this issue, is of interest, illustrated a tremendous splenic enlargement and hyperplasia of lymphatic nodes from a different origin:

J. S., aged 42, Pole, laborer, married. The father is alive and well; the mother died at the age of 28 of causes unknown to the patient. He has been in the United States eight years and is of good habits.

The patient was never sick until two years ago, when he was chilled from exposure, since which time he noticed a slight cough. He was admitted to the German Hospital, April 24, 1905. Two months previous to his admission he noticed that his abdomen was growing larger and that a mass was developing in the left abdominal region. During this time he complained of considerable diarrhea—six to eight stools a day, in some of which blood was present. He also complains of a cough which is worse in the recumbent position, general weakness and at times nose bleed.

*Status Praesens.*—Patient is tall, well-built, of large, bony frame, but with muscles somewhat flaccid. The face shows con-

FIG. 1.

ULLMAN: PSEUDOLEUKEMIA.

siderable pallor, the mucous membranes of the mouth and conjunctivæ are pale. There are many nodular enlargements easily movable corresponding to the lymph nodes; also a hyperplasia of lymphoid tissue of pharynx, pharyngeal tonsils and lingual tonsils. The enlarged lymph nodes are most prominent in the submaxillary, the right and left inguinal and femoral, also in the axillary regions. Some of them can be seen in the illustration. During the period under observation (April 24, 1905, to May 19,

1905,) the pulse which was soft and compressible averaged from 90 to 100, the temperature 99° to 100°, at one time reaching 101°; respirations average 24. The stools are frequent, but contain no blood; at times there has been epistaxis. The abdomen is prominent; a large hard mass corresponding to the spleen fills the entire left upper quadrant of the abdomen. It is regular and its borders are well defined. The liver extends below the free margin of the ribs. There is also a moderate ascites present. There is harsh breathing anteriorly and posteriorly in the apex of the right lung. The heart reveals a systolic bruit at the apex which is transmitted into the axilla and hemic murmurs are heard over the base. There is also some pulsation in the vessels of the neck.

The urine is acid, specific gravity, 1024. There is no sugar, albumin, or casts. The blood examination is as follows: erythrocytes, 2,450,000; leukocytes, 6,000; hemoglobin, 55 per cent.

The white cells show a relative increase of the lymphocytes. The red corpuscles show poikilocytosis with an occasional poikilocytosis, the color index being minus.

From the history of the case at first hand, it was thought to be a case of leukemia, but the blood examination soon determined that it was not, unless, as described later, it was an aleukemic forerunner of leukemia. The blood picture is also suggestive of Banti's disease, which is a cirrhosis of the liver associated with a hyperplasia of the spleen, which may overshadow the cirrhotic condition of the liver. In this disease there may or may not be jaundice and in the terminal stage ascites may appear. It is also suggestive from the blood examination of splenic anemia, but the general lymphatic adenopathy excludes this disease.

The history agrees with Hodgkin's disease in the gradual onset of a widespread hyperplasia of lymphatic structures and spleen, less so of the liver, and tonsils, the anemia of a chloroanemic type, the asthenia, moderate rise of temperature and predisposition to slight hemorrhages.

There are conditions which have the clinical symptoms of pseudoleukemia or Hodgkin's disease, in common, such as tumors of the lymphatics, periods of fever, cachexia and the blood-finding of a normal blood or a chloroanemia. In lymphosarcoma the growths affecting the lymphatics and lymph nodes break through the capsule and there is cohesion of individual groups of lymph nodes reaching into neighboring tissues and organs; the glands being bound by adhesions are often fixed and subjected to ulceration.

Pel (Pseudoleukämie und chronische Rückfallfieber, *Berlin klin. Woch.,* 1887) and Ebstein (Das chronische Rückfallfieber, *Berlin klin. Woch.,* 1887, Nos. 31 and 45-7) described a condition where in addition to the pseudoleukemia there were intervals of fever with periods in which there was no temperature. With the attack of fever the nodes and spleen would enlarge, to decrease in size in the fever-free periods. This symptom complex has been described as chronic recurrent fever (chronische Rückfallfieber). A case resembling this condition was recently seen in consultation with Dr. S. Goldberg, in a young man who has been ailing over one year. There is a moderate anemia. Reds, 3,500,000; hemoglobin, 70 per cent.; whites, 9,000. The patient has a cough and raises some sputum. The examination of sputum shows the presence of tubercle bacilli. The glands of the neck are all very much enlarged; less so of the axilla and the inguinal region. There are periods of temperature followed by days in which there is no temperature.

Sternberg (*Zeit. f. Heilk.*, 19, 1898,) believes pseudoleukemia to be tubercular, for in some cases examined by him the lymph nodes contained tubercle bacilli and in animals inoculated with lymph tissue contracted tuberculosis. Askanazy (Tuberculöse Lymphome unter dem Bilde der Pseudoleukämie; verlaufend Ziegler's Beiträge zur pathologischen Anatomie, 1880, S. 413,) and others also are of this opinion, among whom may be mentioned the work of Joseph Sailer on "The relation of tubercle bacillus and pseudoleukemia (*Philadelphia Medical Journal,* 1902). He believes that pseudoleukemia will ultimately be recognised as being tubercular in character. Ziegler (Ziegler's Pathology) states that we know nothing of the cause of leukemic or pseudoleukemic hyperplasia of the spleen. In some cases the affection has been preceded by some form of injury or infective disease; in other cases there has been nothing of the kind.

The identity of these two diseases is indicated by the fact that they show no anatomical differences and that one form may pass into the other. Stengel (20th Century Practice) also alludes to the fact that the pathological anatomy of both diseases are clearly allied. Indeed, Cohnheim and other German writers have described pseudoleukemia as the aleukemic forerunner (vorstadium) of leukemia.

Among the few recorded cases of the transition from a pseudoleukemia to a terminal leukemia, I would allude to the detailed report of Grover Wende. (A case of lymphatic leukemia apparently developing out of Hodgkin's disease, accompanied by leukemic lesions and pigmentation of the skin, culminating in streptococcus infection.—*American Journal of Medical Sciences,* December, 1901.)

Dock (The influence of complicating diseases upon leukemia. —*American Journal of Medical Sciences,* 1904,) examined the blood changes following or associated with diseases occurring in the course of leukemia, showing that as a result of such intercurrent affections the blood count may fall, and even simulate a condition of leukopenia.

In one case of myelogenous leukemia, complicated by an attack of influenza, the leucocyte count dropped from 367,070 to 5,000, with an increase to 157,000 six weeks after. He mentions many cases from the literature where there was a fall of leucocytes simulating the blood picture of pseudoleukemia from intercurrent infections such as typhoid-like disease, erysipelas, tuberculosis, angina, pneumonia, and the like.

In two cases of acute lymphemia, reported by Fränkel, one of staphylococcus infection, there was a fall from 123,000 to 600 in twelve days. The other colon infection from 220,000 to 1,200 in seven days. In the case of Mueller's of acute lymphemia with streptococci, staphylococci and colon bacilli in the exudate of the pharynx, the leucocytes fell from 109,600 to 6,800 in five days.

In the treatment of our case Fowler's solution was given in gradually increasing doses until evidences of its maximum dose appeared, such as puffiness of the eyelids and edema of the legs. Arsenic has been found an excellent drug in leukemia, a number of observers having noted a fall of leucocytes almost to the normal in that disease. T. McCrae (*British Medical Journal,* 1900,) published a report of a case where not only was there no leukocytosis but no sign of medullary disease in leukemia following the use of arsenic. There are a number of cases recorded of apparent improvement in this affection under the use of the *x*-ray.

In our patient the spleen at times receded only to return again; the lymphatic enlargement remained and he ultimately left the hospital.

### THE SECOND CASE IS ONE OF ELEPHANTIASIS OF THE RIGHT LEG AND THIGH.

Ernest T., aged 25; English; molder; single. He was born in England and came to this country seven years ago. His father died at 37, from a railroad accident. His mother is also dead; age and cause of death is unknown.

The patient had measles when young. Six years ago as a result of infection from his stocking his right little toe became septic and septicemia developed. He was unable to use his leg for about two months. The leg was edematous, painful, was open in several places and discharged pus. The leg since this infection never returned to its original size nor was he able to use it as well as before.

Two years ago while working in the Gowanda State Hospital erysipelas developed in the right leg and he was confined to his bed for two weeks. Following this attack the leg became still

FIG. 2.

ULLMAN: ELEPHANTIASIS.

larger, but he continues to use it in walking, although he soon tires on account of the increased weight of the part.

The patient first presented himself to me at the University of Buffalo Dispensary, complaining of dropsy of one leg. On examination there was not noted the pitting observed in dropsy; the skin was hyperplasic, and but one limb was affected. Some slight vesicles and bullæ were found which discharged a chylous fluid. He states that frequently he notes such formation on the affected limb and following this discharge the leg feels more comfortable. He also notices that if he is on his feet a great deal the leg aches and gets larger, but otherwise there is no pain.

The patient has been at the German Hospital where measurements have been taken of the two extremities:

| | Right Inches. | Left Inches. |
|---|---|---|
| Instep | 12 | 9¾ |
| Ankle | 13¼ | 10 |
| Just above ankle | 14¼ | 8¼ |
| Lower third leg | 15 | 8½ |
| Calf | 17½ | 13 |
| Upper third leg | 17 | 13 |
| Knee | 16 | 13 |
| Lower third thigh | 17 | 13 |
| Mid-thigh | 20½ | 16 |
| Upper thigh | 21 | 18½ |

The length of each extremity is the same.

"Elephantiasis is a chronic endemic and sporadic hyperplasia of the skin and subcutaneous tissue following an inflammatory embolus of the lymph and blood channels and resulting in an inordinate enlargement."—(Sajous's Annual).

Almost any part of the body may be involved, but most frequently the legs and genitalia are affected, although the face, lips and body are less frequently affected.

In the Atlas of Illustrations of Clinical Medical and Surgical Pathology, compiled for the New Sydenham Society, 1904, there is shown an illustration of elephantoid hypertrophy of an upper extremity in a child, due to infection of the finger. In Fox's Atlas of Diseases of the Skin, a case is shown illustrating both legs affected symmetrically.

There is a form of the disease endemic in India and West Indies, Egypt, China, and Arabia, commencing with fever and great pain in the lumbar region, groin and spermatic cords, testes and scrotum. There may also be nausea, vomiting, chills and profuse sweating. The acute attacks disappear leaving the extremities larger than before. The enlargement may be so great as to obliterate the natural shape of the limb. In the endemic cases the disease is due to the occlusion of the larger lymphatic vessels by the filaria sanguinis hominis. In our case the elephantoid enlargement followed an erysipelatous infection. The patient left the hospital unimproved.

### RACHITIS WITH MARKED THORACIC DEFORMITY.

S. M., aged 14 months; Italian. The parents are badly nourished and live in a crowded tenement. The child is breast fed. The baby has been sick for three months, developing measles, followed by lobular pneumonia. The baby is anemic and underfed, and a well marked example of rachitis. The anterior fontanelle is patent; the frontal boses are prominent. The head takes on a square and brachycephalic shape.

The epiphyses of the ulna and radius are thickened. The interesting part of our case is the extreme deformity of the chest. The ribs yielding to atmospheric pressure and the action of the diaphragm resulting from the dyspnea due to the pulmonary disease shown typically the depressions, laterally, driving in the lungs

FIGS. 3 AND 4.

ULLMAN: RACHITIS.

and pushing back the heart, producing posteriorly a kyphosis and anteriorly pushing the sternum forward, producing the chicken breast (pectus carinatum).

The lateral depression of the ribs affects only the middle ones, for the upper ribs are short and better protected by thick muscles, while the lower ribs are more movable. There is general improvement noted with inunctions of cod liver oil, fresh milk, and the use of hypophosphites.

I desire to express my thanks to Dr. Robert C. Mehnert of the German Hospital for the histories of two cases, and to Dr. Bethune for the last photograph.

400 FRANKLIN STREET.

## Figures and Tables

**Fig. I. f1:**
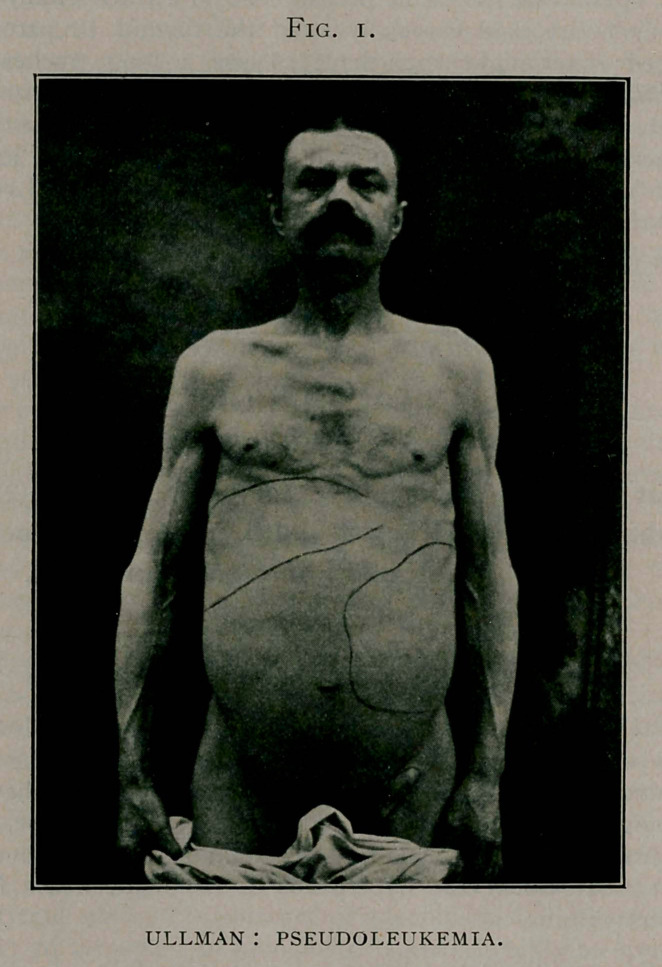


**Fig. 2. f2:**
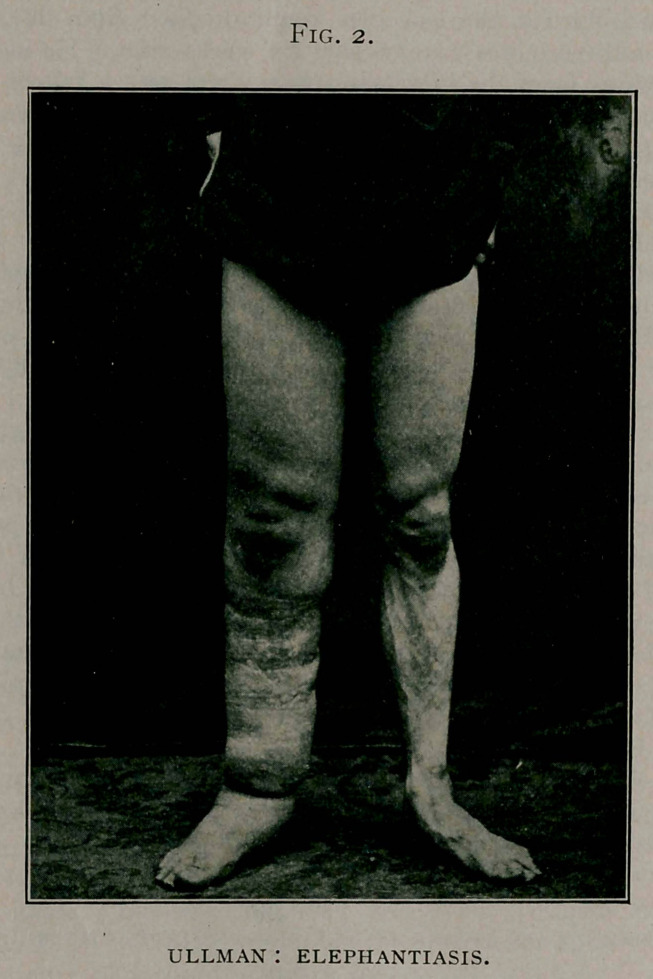


**Figs. 3. and 4. f3:**